# Hypothyroidism among Type 2 Diabetic Patients Visiting Outpatient Department of Internal Medicine of a Tertiary Care Centre: A Descriptive Cross-sectional Study

**DOI:** 10.31729/jnma.8130

**Published:** 2023-04-30

**Authors:** Biju Shrestha, Chandra Kala Rai

**Affiliations:** 1 Department of Physiology, Kathmandu Medical College and Teaching Hospital, Duwakot, Bhaktapur, Nepal

**Keywords:** *prevalence*, *thyroid dysfunction*, *type 2 diabetes mellitus*

## Abstract

**Introduction:**

Diabetes-thyroid relationship is said to be bidirectional. Insulin resistance and hyperinsulinemia in type 2 diabetes mellitus increase free thyroxine but decrease free triiodothyronine and thyroid-releasing hormone synthesis. Thyroid dysfunction may in turn impose an adverse effect on glucose metabolism in type 2 diabetes mellitus. Undetected thyroid dysfunction can worsen glycemic control and predispose type 2 diabetes mellitus patients to cardiovascular and other diabetes-related complications. Recognition and timely treatment of thyroid dysfunction in type 2 diabetes mellitus patients can delay diabetic complications. The aim of this study was to find out the prevalence of hypothyroidism among type 2 diabetic patients visiting the outpatient Department of Internal Medicine of a tertiary care centre.

**Methods:**

A descriptive cross-sectional study was conducted from 17 April 2021 to 5 September 2021 after obtaining ethical approval from the Institutional Review Committee (Reference number: 130120202). A total of 384 type 2 diabetic subjects were recruited for the study. Convenience sampling method was used. Point estimate and 95% CI were calculated.

**Results:**

Among 384 patients, the prevalence of hypothyroidism was found in 127 (33.07%) (28.3637.78, 95% Confidence Interval). Among them, 56 (44.09%) were male and 71 (55.90%) were female. The mean age was 55.17±7.53 years.

**Conclusions:**

The prevalence of hypothyroidism was higher than in the other studies done in similar settings.

## INTRODUCTION

Thyroid dysfunction and Diabetes Mellitus (DM) are the two endocrine disorders commonly encountered in the adult population nowadays and they tend to coexist.^1^ Patients with type 2 diabetes mellitus (T2DM) have a high tendency to develop thyroid dysfunction. Insulin resistance as seen in T2DM plays a major role in the development of thyroid dysfunction in such patients.^2^ Studies have found a significantly higher prevalence of thyroid disorders in T2DM patients.^3^

Unrecognized thyroid dysfunction may impair metabolic controls in patients with T2DM and further worsen diabetic complications. Recently, there is a rising prevalence of T2DM in developing countries including Nepal. The thyroid profile in the Nepalese population with DM has not been studied earlier.^4^ Recognition and timely treatment of thyroid dysfunction in T2DM patients can delay diabetic complications.

The aim of this study was to find out the prevalence of hypothyroidism among T2DM patients visiting the outpatient Department of Internal Medicine of a tertiary care centre.

## METHODS

This descriptive cross-sectional study was conducted among T2DM patients attending the Department of Medicine who were advised for thyroid function tests in the Pathology lab, Kathmandu Medical College and Teaching Hospital, from 17 April 2021 to 5 September 2021. Ethical apprval was obtained from the Institutional Review Committee (IRC) of the same institution (Reference number: 130120202). Patients aged 40-70 years, with T2DM for more than 5 years, without insulin and other debilitating diseases were included. Gender, age (years), and duration of T2DM (years) were assessed. Convenience sampling was done and the sample size was calculated using the following formula:


$$\begin{array}{ll} \text{n} & ={\text{Z}}^{2}\times \frac{\text{p}\times \text{q}}{{\text{e}}^{\text{2}}}\\ & =1.96^{2}\times \frac{0.50\times 0.50}{0.06^{2}}\\ & =267 \end{array}$$

Where,

n = minimum required sample size
Z = 1.96 at 95% Confidence Interval (CI)
p = prevalence taken as 50% for maximum sample size calculation
q = 1-p
e = margin of error, 6%

The calculated samples size was 267. Adding 10% non-response rate the calculated sample size is 297. However, a total of 384 sample size was taken.

Venous blood samples were collected and assessed for thyroid function tests. The thyroid function test was done by using Chemi Lumination Immuno Assay (CLIA) and Ultra-Sensitive CLIA method. Any deviation from the normal range is considered thyroid dysfunction. Normal reference range for TSH (0.3-3.5 mU/l) Free T4 (10-25 pmol/L) and Free T3 (3.5-7.5 pmol/L).^5^ Based on the given normal reference range, we have classified clinical hypothyroidism if TSH is high above normal range and FT4 and FT3 below normal range; Similarly, subclinical hypothyroidism if TSH is high above normal range and FT4 and FT3 in normal range. Written informed consent was obtained from all of the included patients.

Data were analyzed using IBM SPSS Statistics version 25.0. Point estimate and 95% CI were calculated.

## RESULTS

Among 384 T2DM patients, the prevalence of hypothyroidism was 127 (33.07%) (28.36-37.78, 95% CI). The mean age of patients was 55.17±7.53 years. Out of these, 54 (14.06%) had clinical hypothyroidism and 73 (19.01%) had subclinical hypothyroidism among patients with T2DM (Table 1).

**Table 1:** Gender-wise distribution of hypothyroidism (n = 127)

Gender	Clinical n (%)	Subclinical n (%)
Male	21 (16.53)	35 (27.56)
Female	33 (25.98)	38 (29.92)

Among patients with clinical hypothyroidism, patients with age >55 years was present in 8 (6.29%) male (Table 2).

**Table 2:** Age and genderwise distribution (n = 127).

Parameters	Age (years)	Male n (%)	Female n (%)
Clinical	<55	8 (6.29)	14 (11.02)
	>55	13 (10.23)	19 (14.96)
Subclinical	<55	18 (14.17)	11 (8.66)
	>55	17 (13.38)	27 (21.26)

Patients with T2DM for more than 10-year duration had 65 (78.30%) prevalence of subclinical hypothyroidism (Table 3).

**Table 3:** Duration of diabetes among hypothyroid (n= 127).

Duration (years)	Clinical n (%)	Subclinical n (%)
<10	9 (7.09)	65 (51.18)
>10	45 (35.43)	8 (6.29)

A total of 42 (33.07%) of patients with subclinical hypothyroidism and 61 (48.03%) of hypothyroidism patients had HBA1C >6.4 (Table 4).

**Table 4:** Hypothyroidism in relation to HbA1C (n = 127).

HbA1C	Clinical n (%)	Subclinical n (%)
<5.7	4 (3.14)	3 (2.36)
5.7-6.4	8 (6.29)	9 (7.08)
>6.4	42 (33.07)	61 (48.03)

## DISCUSSION

Among 384 T2DM patients, the prevalence of hypothyroidism was 33.07% in our study. In this study, 14.06% of T2DM patients had clinical hypothyroidism and 19.01% of T2DM patients had subclinical hypothyroidism. There is significant evidence among T2DM patients suggesting that increased age and gender, are associated with an increased risk of developing hypothyroidism. The prevalence of subclinical hypothyroidism is known to increase with age.^5^ We found a high prevalence of subclinical hypothyroidism (19.01%) in T2DM patients. Different studies found prevalece of hypothroidism (26.5%), (20%), (10.7%), (14%) and (18%) which is similar to our sudy.^4,6-9^ The present study also documented 14.06% of clinical hypothyroidism in T2DM patients which was a similar finding to the study (13.9%).^2,10^ Our study found a high prevalence of subclinical hypothyroidism in female T2DM patients than in male T2DM patients. This finding was in accordance with these studies.^11,12^ Another study done in Saudi Arabia also documented similar results.^13^

Furthermore, a cross-sectional observational study among T2DM patients in India found a significantly increased risk of hypothyroidism in the elderly > 65 years.^5^ The mean ageof patients was 55.17±7.53 years. We found a higher prevalence of subclinical hypothyroidism in elderly T2DM aged above 55 years. Our study found that T2DM patients with a duration of diabetes less than 10 years had subclinical hypothyroidism and those with a diabetes duration of more than 10 years had clinical hypothyroidism. Thyroid dysfunction may affect glucose metabolism in T2DM. Changes in serum TSH were found to be correlated with changes in glycated haemoglobin (HbA1c).^5^ The present study documented a significant association between subclinical hypothyroidism in T2DM. However, a study showed that thyroid dysfunction was not linked to the duration of diabetes, and glycosylated haemoglobin.^7^

Diabetes-thyroid relationship is said to be bidirectional. Insulin resistance and hyperinsulinemia in T2DM increase FT4 but decrease FT3 and TRH synthesis.^14^ Thyroid dysfunction may in turn impose an adverse effect on glucose metabolism in T2DM.^5^ Hyperthyroidism impairs glycemic control and hypothyroidism may cause hypoglycaemia. Both forms of thyroid dysfunction may exaggerate cardiovascular risk and worsen diabetic complications in T2DM. Many review and meta-analysis studies have reported cardiovascular and diabetic complications in T2DM with thyroid dysfunction.^1^

Limitations of our study were, it is a hospital-based, single-centred study and not a cross-sectional population study. The finding of this study may not exactly represent the general population at a large scale.

## CONCLUSIONS

The prevalence of hypothyroidism among the patients with T2DM visiting outpatient department of internal medicine was higher than in other studies conducted in similar settings.
